# A case of disseminated neurocysticercosis

**DOI:** 10.4103/0972-2327.78054

**Published:** 2011

**Authors:** D. Saranya, M. Jawahar, K. Bhanu

**Affiliations:** Department of Neurology, Institute of Neurology, Madras Medical College and Government General Hospital, Chennai - 600 003, India

## Introduction

Neurocysticercosis is the most common parasitic infection of the central nervous system. However, disseminated cysticercosis is rare.[1] We present a case of disseminated neurocysticercosis with characteristic imaging findings. A 60-year-old male presented with a history of complex partial seizures and frontal headache for two months. There was no history of limb weakness, diplopia, sensory deficits, bladder or bowel disturbances. There was no history of fever or trauma. General examination revealed multiple pea-sized nodules. The central nervous system examination and fundus examination were normal. The patient did not give consent for biopsy of the subcutaneous nodules.

**Figure 1:**
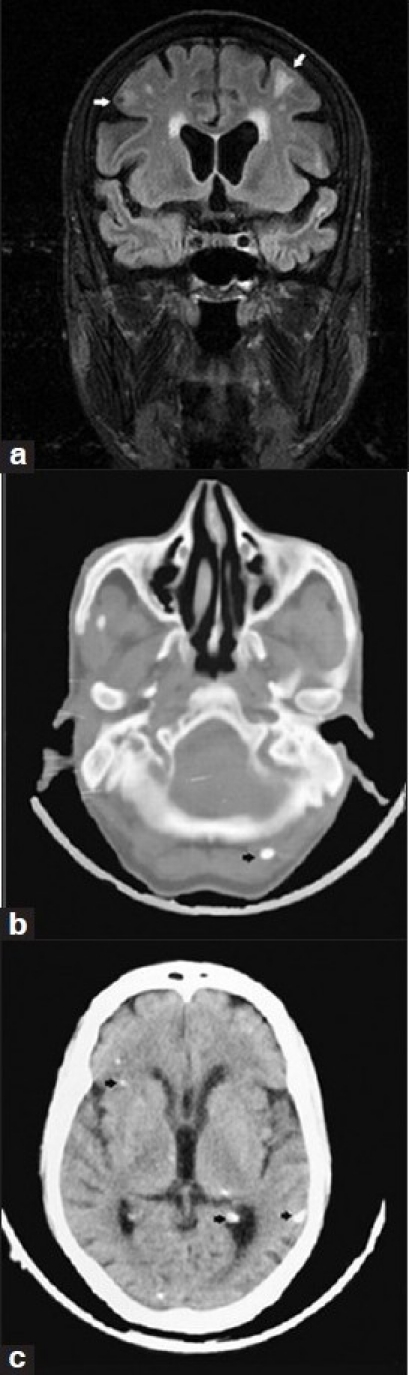
(a) Magnetic resonance imaging of the brain showing a hypodense lesion surrounded by perilesional edema in the right parietal lobe and edema in the left parietal lobe, (b) The computerised tomography scan of the brain showing a calcified lesion in the soft tissue of the scalp and (c) multiple calcified lesions located bilaterally in the brain parenchyma

**Figure 2:**
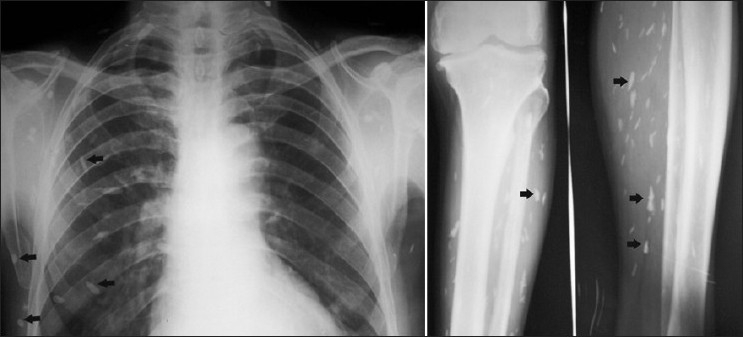
The chest radiograph showing multiple cigar-shaped calcifications in the subcutaneous plane (left arrows). The radiograph of the calf showing multiple cigar-shaped calcified lesions arranged in the subcutaneous plane and parallel to the muscle fibres in the intramuscular plane (centre and right arrows).

A computed tomography (CT) scan of the brain showed multiple calcified lesions located bilaterally in the supratentorium and in the infratentorium. A calcified lesion was also seen in the soft tissue of the scalp. The magnetic resonance imaging (MRI) of the brain showed hypodense lesions surrounded by peri-lesional edema in the frontal and parietal lobes[Figure 1]. The plain radiograph of the calf showed multiple cigar-shaped calcified lesions arranged parallel to the muscle fibres in the intramuscular plane and in the subcutaneous plane. The chest radiograph also revealed multiple cigar-shaped calcifications in the subcutaneous plane [Figure 2]. These characteristic imaging findings in a patient from an endemic area and a compatible clinical picture points to the diagnosis of systemic disseminated cysticercosis.[2]

The CT picture in neurocysticercosis depends on the number, location, and stage of the lesions.[3] In the vesicular stage (viable cysts), the cysts characteristically appear circumscribed and hypodense and do not enhance after contrast administration. Most of these lesions have in their interior an eccentric hyperdense nodule representing the scolex. On an MRI scan, vesicular cysts appear with signal properties similar to those of cerebrospinal fluid (CSF) in both T1- and T2-weighted images. The scolex is usually visualized within the cyst as a high-intensity nodule. In the colloidal stage, which represents a dying cyst, there is a ring-enhancing lesion surrounded by white matter edema. On an MRI scan, the wall of the cyst becomes thick and hypointense and there is marked perilesional edema. In the next nodular granular stage, the lesions are homogenously enhancing and they ultimately calcify. The calcified stage, representing a dead parasite, appears as a hyperdense lesion on noncontrast CT scans. The CT picture of our patient depicts the calcified stage of the cysticercal larvae. These lesions are usually not visualized with MRI. Although these calcified nodules are usually without perilesional edema, recent studies have shown that perilesional edema is common and associated with seizures in patients with calcified neurocysticercosis.[4,5] Plain radiographs of the soft tissue involved typically show multiple cigar-shaped calcified lesions arranged parallel to the muscle fibres in the intramuscular plane and in the subcutaneous plane.
